# The paradox of mechanical ventilation: life-saving, injurious, and pro-inflammatory – lung protection during general anesthesia with neuromuscular blockade

**DOI:** 10.1016/j.bjane.2026.844822

**Published:** 2026-09-03

**Authors:** Jean-Jacques Rouby, Jean-Michel Constantin

**Affiliations:** a Research Department DMU DREAM, Clinical Research Group 29 (GRC 29), Sorbonne University of Paris, France; b Multidisciplinary Intensive Care Unit, Department of Perioperative Medicine, La Pitié-Salpêtrière Hospital, Paris (75013), Assistance-Publique Hôpitaux de Paris (AP-HP)

In this issue of the journal, the Brazilian Society of Anesthesiology releases Consensus Recommendations on Perioperative Mechanical Ventilation Strategies.^1^ This Editorial analyzes and summarizes the different steps that led to these recommendations since the 1950s, with a specific attention to tidal volume (TV) and recruitment maneuvers.

## From the early 1950s to the 1980s: the use of mechanical ventilation and the rationale for delivering tidal volumes ≥ 10 mL.kg^-1^

In the mid-20th century, two major medical innovations revolutionized surgery and medicine: general anesthesia and mechanical ventilation. Both are tightly linked: the administration of anesthetic agents for inducing sleep, opioids for controlling pain and muscle relaxants for ensuring a quiet operating theater implies mechanical ventilation for supporting gas exchange. A critical step was the 1952 poliomyelitis epidemic in Copenhagen: the life of thousands of patients dying from muscle paralysis-induced respiratory failure was saved by inserting a tube in the trachea and squeezing intermittently an air oxygen mixture into the lungs.^2^ Two-hundred and fifty medical students and 260 nurses were hired for ensuring 24-hour manually gas insufflation until recovery from respiratory muscles paralysis. Mortality rate decreased from 87 to 25%. Critical Care Medicine was born.^3^ For the next 20 years, artificial respirators providing mechanical ventilation (MV) were manufactured, offering the possibility of individualizing ventilator settings: tidal volume (TV), respiratory rate, inspiratory time, positive end-expiratory pressure (PEEP), and sighs (recruitment maneuvers). The early introduction of large screens displaying flow and pressure waveforms and the possible monitoring of physiologic respiratory parameters allowed to personalize MV.^4^

With the widespread development of intraoperative MV in the 1960s, anesthesiologists noticed that anesthetized patients became hypoxemic soon after anesthetic induction. In a physiologic study performed in 22 patients undergoing upper abdominal surgery and mechanically ventilated with physiologic TV, Bendixen and co-workers demonstrated that radiologic atelectasis occurred in dependent lung regions, causing a right-to-left shunt and a 22% average drop in PaO_2_ despite the use of 100% oxygen.^5^ Lung collapse and hypoxemia were reversed by three consecutive vital capacity inflations.^6^ In series of patients anesthetized for various types of surgery, mechanical ventilation using TV ≥ 10 mL.kg^-1^ of ideal body weight also reversed arterial hypoxemia.^7^, ^8^, ^9^ These studies established the rationale for using TV of 10-12 mL.kg^-1^ with periodic sighs in Anesthesiology and Critical Care for the next 40 years. Between 1970 and 2010, generations of residents in Anesthesiology and Critical Care were trained to deliver extra-physiologic TV, to control PaCO_2_ by setting respiratory frequency between 10 and 15 bpm, and target FiO_2_ to get an oxygen saturation ranging between 95 and 100%.

## The period 1980-2000: mechanical ventilation in patients with acute respiratory distress syndrome and the rationale for delivering tidal volumes ≤ 6 mL.kg^-1^

The first warning about the dangers of delivering TV ≥ 10 mL.kg^-1^ came to the forefront in the mid-1980s. A high-permeability type pulmonary edema was produced in normal rats after a 20-minute period of MV delivering a TV of 45 mL.kg^-1^ at a peak inspiratory pressure of 45 cmH_2_O.^10^ Alveolar flooding by an albumin-rich edema resulted from a disruption of the alveolar-capillary barrier, the cause of which was excessive TV (volutrauma) rather than excessive airway pressure (barotrauma).^11^ At first glance, these experimental conditions seemed extravagant, without human equivalent and considered lacking clinical relevance. However, the understanding that these experiments were relevant to critically ill patients with severe Acute Respiratory Distress Syndrome (ARDS) was rapidly acknowledged.

**Figure 1:**
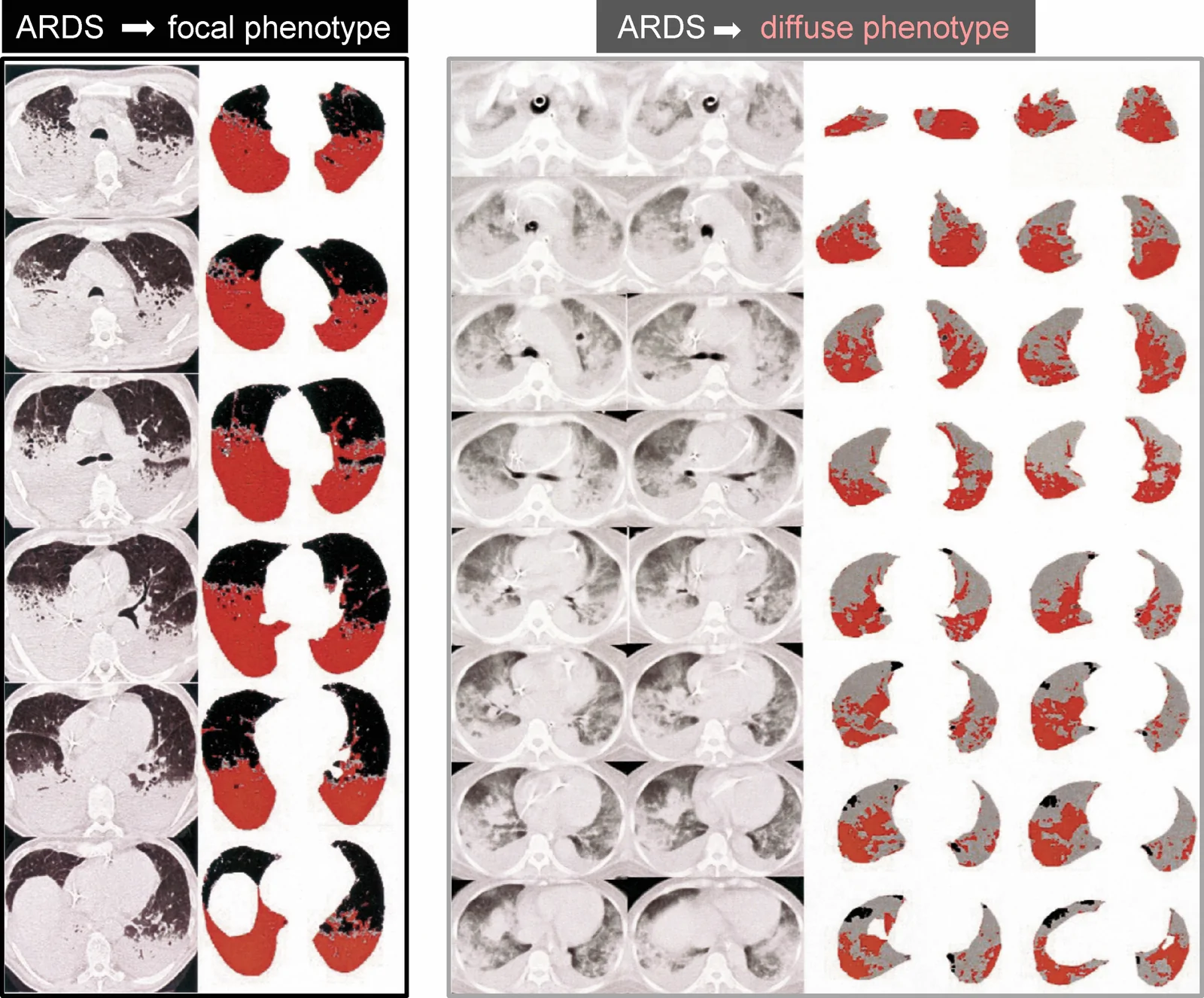
Acute Respiratory Distress Syndrome (ARDS) phenotypes based on the distribution of aeration loss (lung morphology). (A) Focal ARDS phenotype. Computed tomographic (CT) scan was performed in a 61 yr-old patient with ARDS caused by aspiration pneumonia in the postoperative period of a hip replacement. The color-coded CT sections show the distribution of normal aeration in black [CT attenuations ranging between -900 and -500 Hounsfield units (HU)], partial loss of aeration in gray (CT attenuations ranging between -500 and -100 HU) and total loss of aeration in red (CT attenuations ranging between -100 and +100 HU). Dependent parts of upper, middle and lower lobes are non aerated whereas nondependent parts show normal aeration. The proportion of poorly aerated lung is small. (B) Diffuse ARDS phenotype. CT scan was obtained in a 50 yr-old patient with ARDS caused by *Pneumocystis carinii*. Poorly and non aerated lung regions are predominant and homogeneously distributed. The proportion of normally aerated lung is small and essentially present in nondependent parts of the right middle lobe. Reproduced from reference^13^ with permission of the publisher.

The ARDS lung is characterized by a massive loss of lung aeration resulting from alveolar flooding by protein-rich edema, infiltration of alveolar space by inflammatory cells, diffuse lung inflammation and bronchiolar collapse/obstruction. Of note, computed tomography images showed that the aeration loss was often heterogeneously distributed (Fig. 1): in a high proportion of ARDS patients lying in the supine position, dependent lung regions (lower lobes) were non aerated whereas nondependent regions (upper and middle lobes) remained partially or fully aerated, defining the focal ARDS morphology.^12^ In other ARDS patients the aeration loss was more homogeneously distributed, involving dependent and non dependent regions, defining the diffuse ARDS morphology.^12^^,^^13^ In very severe forms of ARDS, the aerated lung volume was found to be reduced to less than 25%, and "functional lung" dimensions become close to the size of a "baby lung".^12^^,^^14^ It was then understood that TV of 10-12 mL.kg^-1^ administered to such ARDS lungs were equivalent to TV of 40-50 mL.kg^-1^ delivered to healthy lungs, exposing the "functional" ARDS lung to volutrauma. In 2000, the negative impact of large TV on ARDS outcome was demonstrated by the ARDS network randomized controlled trial,^15^ where traditional ventilation delivering a TV of 12 mL.kg^-1^ was compared to a low-TV ventilation delivering a TV of 6 mL.kg^-1^. The trial was stopped after the enrollment of 861 patients because mortality was 20% lower in the group treated with low TV (31% vs. 40%, p = 0.007). From the 2000s, TV ≤ 6 mL.kg^-1^ of ideal body weight became the reference for the ventilation of ARDS patients. However, a TV of 6 mL.kg^-1^ does not always protect against ventilator-induced lung injury.^16^ There is a basic reason for that: scaling the TV to the healthy lung size does not fit the conditions of the ARDS lung, whose aerated part – the "functional" lung – is much smaller than the total lung volume. The ARDS lung is stiff because small and the reduction of respiratory compliance (Crs) is correlated to the volume of the functional lung.^17^ Therefore, it appears logical to scale the TV to the respiratory compliance rather than to the lung size. The ratio TV/Crs defines the driving pressure (mmHg), which is equal to plateau pressure minus PEEP in mechanically ventilated patients fully assisted by the ventilator.^18^ In the mid-2000s, it was demonstrated that a driving pressure > 10 cmH_2_O is highly predictive of excess mortality in ARDS patients.^18^^,^^19^ The safe TV, expressed in mL.kg^-1^, is the TV which provides the lowest driving pressure (10-15 cmH_2_O) and enough CO_2_ elimination to achieve a ph ≥ 7.15.

**Figure 2:**
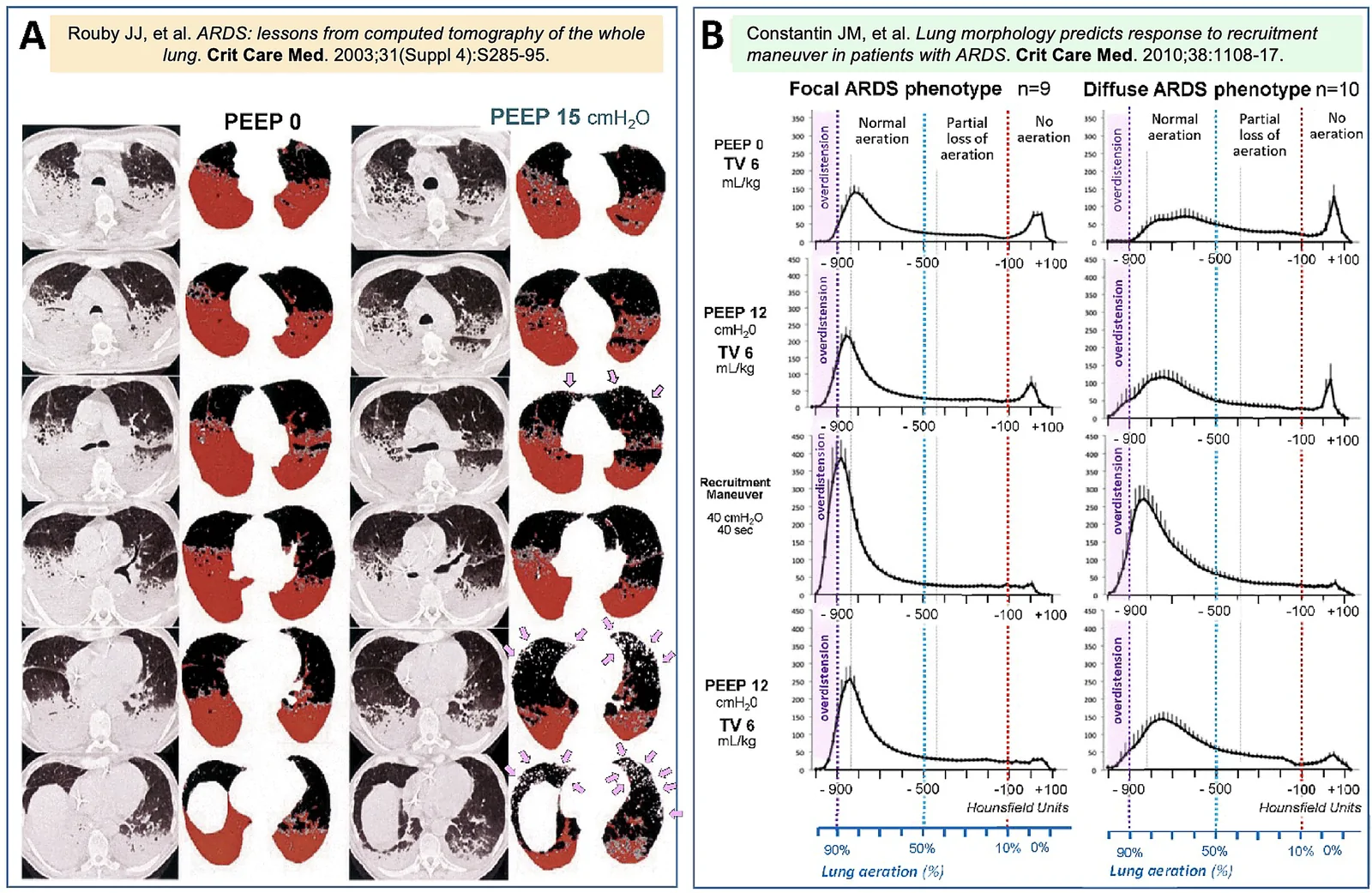
Positive end-expiratory pressure (PEEP) greater than 10 cmH_2_O and high-pressure recruitment maneuvers recruit some parts of the lung and overdistend simultaneously some other parts. (A) Effect of high PEEP in focal ARDS phenotype. Computed tomographic (CT) scan obtained in a 61 yr-old patient with ARDS caused by aspiration pneumonia in the postoperative period of a hip replacement. CT sections were obtained at end expiration without PEEP and after PEEP 15 cmH_2_O. The color-coded CT sections show the distribution of overdistension in white [CT attenuations lower than -900 Hounsfield units (HU)], normal aeration in black (CT attenuations ranging between -900 and -500 HU), partial loss of aeration in gray (CT attenuations ranging between -500 and -100 HU) and total loss of aeration in red (CT attenuations ranging between -100 and +100 HU). At PEEP 15, overdistended lung areas are visible in nondependent parts of upper and middle lobes (purple arrows) and recruitment is visible in dependent parts of lower lobes (red color replaced by gray or black color). Reproduced from reference^13^ with permission of the publisher. (B) Effect of high PEEP and high-pressure recruitment maneuver in focal (n = 9) and diffuse (n = 10) ARDS phenotypes. Volumic distribution of CT attenuations (Y-axis) is expressed in Hounsfield Units (X-axis)**.** The Hounsfield scale is based on the gas/tissue ratio and reflect the aeration expressed in % (blue X-axis at the bottom of the figure). All patients are receiving a TV of 6 mL.kg^-1^. Four conditions are studied: PEEP 0, PEEP 12 cmH_2_O, high-pressure recruitment maneuver (40 cmH_2_O for 40 sec), and again PEEP 12 cmH_2_O. In all patients, high PEEP and recruitment maneuver recruited non aerated lung regions and overdistended previously aerated lung regions. PEEP-induced overdistension was 327 ± 143 mL in focal ARDS and remained very low in diffuse ARDS, 49 ± 68 mL. Reproduced from reference^22^ with permission of the publisher.

**Figure 3:**
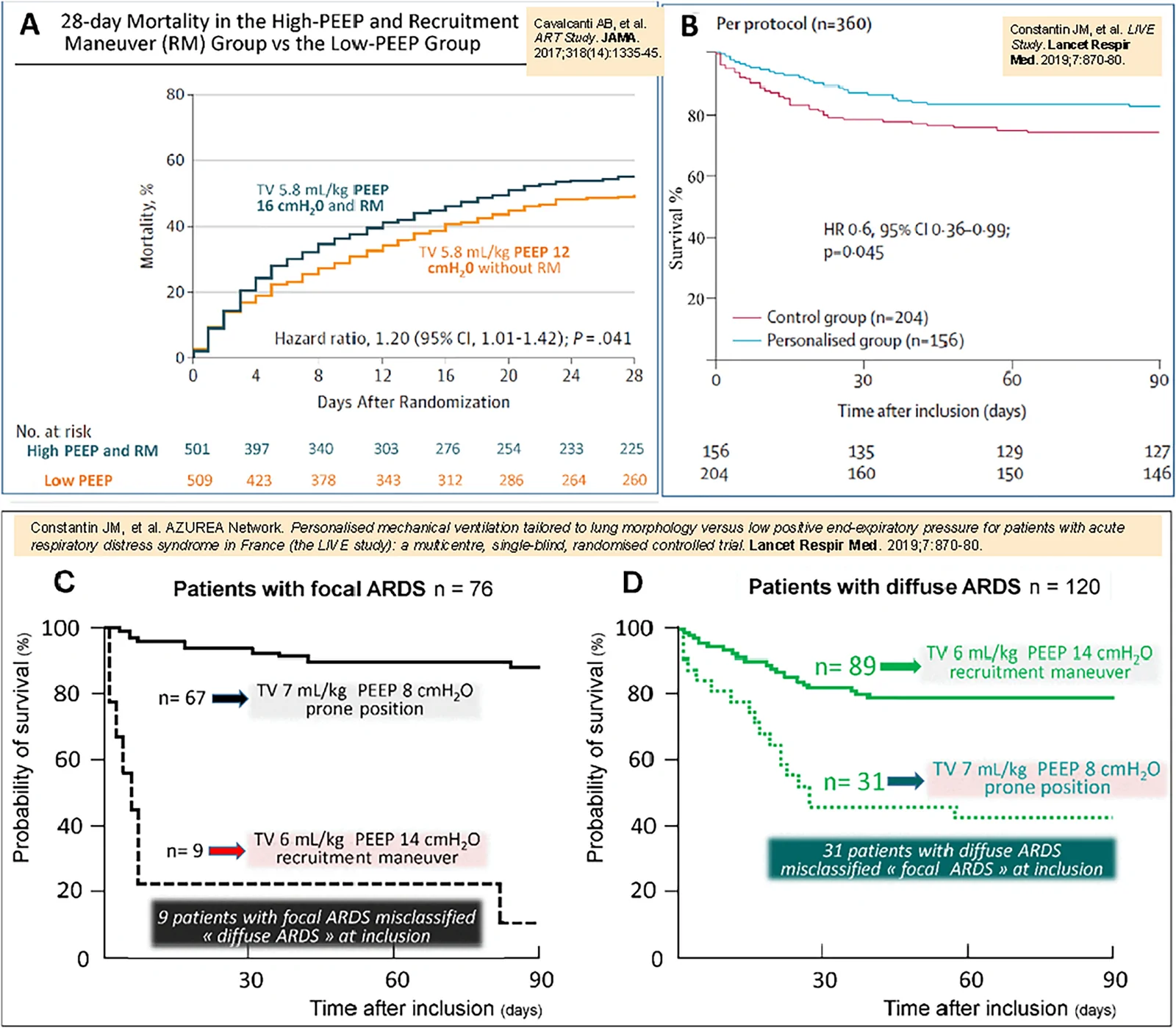
Effects of high positive end-expiratory pressure (PEEP) and high-pressure recruitment maneuver on ARDS patients’ outcome. (A) In the ART multicenter randomized controlled trial, 1010 patients were randomized to high PEEP (16 cmH_2_O) with high-pressure recruitment maneuvers vs moderate PEEP (12 cmH_2_O) without recruitment maneuver. All patients received TV of 5.8 mL.kg^-1^. Compared with the moderate PEEP strategy, the high-PEEP strategy increased 28-day mortality, decreased the number of ventilator-free days, increased the risk of pneumothorax requiring drainage and the risk of barotrauma. There were no significant differences in the length of ICU stay, length of hospital stay, ICU mortality, and in-hospital mortality. Reproduced from reference^24^ with permission of the publisher. (B) Effect of personalized ventilatory support according to ARDS phenotype. At inclusion in the LIVE multicenter randomized controlled trial, ARDS morphology was classified into focal or diffuse (nonfocal) phenotype. 156 patients were correctly classified (76 focal and 120 diffuse) and 40 were misclassified (9 focal patients were classified as diffuse ARDS and 31 diffuse patients were classified as focal ARDS). In the personalised group, patients with focal ARDS were treated with low PEEP, no recruitment maneuver, and prone position and patients with diffuse ARDS were treated with high PEEP and high-pressure recruitment maneuvers. In the per protocol analysis (156 patients correctly classified), 28-day and 3-month survival was higher when the respiratory support was tailored to lung morphology. Reproduced from reference^26^ with permission of the publisher. (C) Among the 76 patients with focal ARDS, 9 were misclassified as diffuse and were treated with high PEEP and high-pressure recruitment maneuvers. Survival at day 28 was reduced to 20%. (D) Among the 120 patients with diffuse ARDS, 31 were misclassified as focal and were treated with low PEEP, no recruitment maneuver, and prone position. Survival at day 28 was reduced to 40%. Reproduced from reference^26^ with permission of the publisher.

It also became evident that in patients with focal ARDS, PEEP- and recruitment maneuver-induced recruitment was inextricably linked to overdistention (Fig. 2).^20^, ^21^, ^22^ These data seriously questionned the safety and the validity of the "open lung" concept.^23^ As shown by the ART randomized controlled trial, an "open lung strategy",^24^ including a mean PEEP of 17 ± 4 cmH_2_O (titrated according to the best Crs^25^) and intermittent recruitment maneuvers, increased mortality when applied indiscriminately to patients fulfilling ARDS criteria.^24^ The LIVE randomized controlled trial then demonstrated that a ventilator strategy misaligned with lung morphology substantially increases mortality (Fig. 3): patients with focal ARDS treated by an open lung strategy (PEEP 14 ± 3 cmH_2_O, titrated to reach a P_plat_ of 30 cmH_2_O) had a mortality of 80% versus 20% when treated by a moderate PEEP (5-10 cmH_2_O) combined with prone positioning.^26^

## The period 2000-2026: mechanical ventilation in patients with healthy lungs and the rationale for delivering tidal volumes ≤ 8 mL.kg^-1^ during general anesthesia

The understanding of benefits and harms of MV in critically ill patients incited anesthesiologists, who play a major role in critical care medicine, to reconsider their practice. MV in anesthetized patients without lung injury and MV in ARDS patients have similarities and differences. Both are associated with loss of lung aeration, but the degree of aeration loss differs markedly between the two.

**Figure 4:**
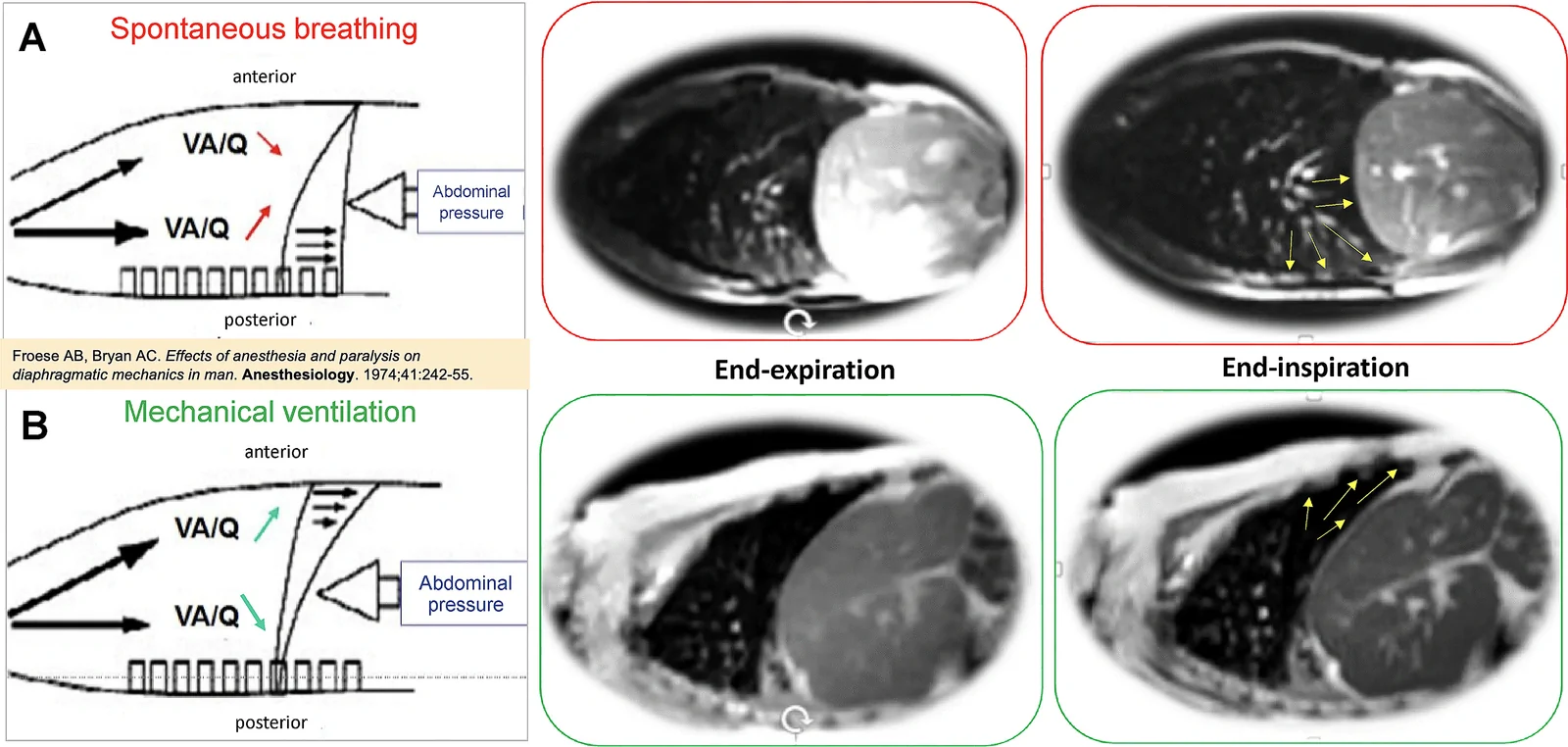
Effects of general anesthesia, muscle relaxation and mechanical ventilation on lung volume and diaphragm activity. (A) In spontaneous breathing, the increase in lung volume during inspiration results from the contraction of inspiratory muscles. The contraction of intercostal muscles increases the antero-posterior dimension of the thorax whereas the contraction of the diaphragm increases its cephalo-caudal dimension. The diaphragm contraction predominates in postero-basal regions and counteracts the abdominal pressure and the cardiac weight (yellow arrows). Ventilation perfusion ratio (VA/Q) increases markedly in dependent and paravertebral lung regions. (B) General anesthesia and muscle relaxation eliminate respiratory muscles activity. During mechanical ventilation, the TV enters the respiratory system as a result of the driving pressure. It is directed towards anterior and nondependent regions, the more compliant parts of the lung (yellow arrows). During inspiration, posterior parts of the diaphragm do not move anymore caudally, the abdominal pressure and the cardiac weight are not counteracted and compression atelectasis occurs, decreasing VA/Q to 0 in dependent lung regions (atelectasis). Lung images are obtained from dynamic magnetic resonance imaging.

Massive in ARDS, aeration loss in patients undergoing general anesthesia is more limited, reaching 5% of the lung volume in general surgery,^27^ and 25% in cardiac surgery with cardiopulmonary bypass.^28^ Atelectasis of dependent regions of lower lobes appears immediately following anesthetic induction,^27^ and is caused by the passive mobilization of the relaxed diaphragm by the gas flow coming from the ventilator (Fig. 4).^29^ The compression of lower lobes by the heart^30^^,^^31^ and the abdominal content contributes to lung collapse. Because aeration loss during general anesthesia was found limited, delivering TV 10-12 mL.kg^-1^ was not considered to expose the lungs to volutrauma like observed in ARDS. However, from the mid-2000s onwards, intraoperative TV were progressively reduced to reach 8 mL.kg^-1^ in 2013.^32^

The rationale for delivering low TV during anesthesia came from studies performed in ventilated critically ill patients without ARDS. A meta-analysis of 2822 ventilated critically ill patients enrolled in 20 studies, 15 of which were randomized controlled trials, provided evidence that delivering 5-8 mL.kg^-1^ TV prevented the onset of ARDS, atelectasis, and pulmonary infection, reduced the length of intensive care unit stay and decreased mortality by 36%.^33^ In other words, low TV ventilation in the critically ill free of lung injury prevented respiratory complications and reduced mortality. By analogy with mechanical ventilation in ARDS patients,^34^ two mechanisms were implicated to explain how high TV could predispose to respiratory complications in critically ill patients with healthy lungs: 1) the "lung biotrauma", defined by the release of pro-inflammatory cytokines by alveolar macrophages, pulmonary epithelial and endothelial cells submitted to positive pressure MV-induced mechanical stretch; 2) the "two hit hypothesis": MV administered to patients without acute lung injury, generates a pulmonary inflammation, akin to priming, that is limited, contained and restrained; infectious challenge or any other inflammatory agression act as secondary hits, that generate an inflammatory response which escapes regulatory mechanisms and may lead to respiratory complications and death.^34^

Biotrauma was initially identified in an isolated rat model.^35^ MV-induced "lung biotrauma" was evidenced first in ARDS patients,^36^ then in critically ill patients without lung injury,^37^ and last but not least in patients with healthy lungs undergoing major thoracic or cardiac surgery.^38^^,^^39^ In the randomized controlled trials performed in ventilated critically ill patients with^36^ and without ARDS,^37^ pro-inflammatory cytokins (TNF-α, IL-6, IL-8, and IL-1β), were found initially elevated in the bronchoalveolar lavage fluid in all patients. They remained stable after a period of MV delivering low TV (5 and 8 mL.kg^-1^) and markedly increased when 10-12 mL.kg^-1^ TV were used. In the 2 randomized controlled trials performed in anesthetized patients undergoing thoracic or cardiac surgery, pro-inflammatory cytokins [TNF-α, IL-6, IL-8, and soluble intercellular adhesion molecule (sICAM-1)], were found initially low in the bronchoalveolar lavage fluid. TNF-α, IL-8, and sICAM-1 increased after one-lung ventilation, but bronchoalveolar concentrations were lower in patients receiving a TV of 5 mL.kg^-1^ vs 10 mL.kg^-1^.^38^^,^^39^ In patients undergoing cardiac surgery, IL-6 and IL-8 were found initially low in the bronchoalveolar lavage fluid. They increased markedly after cardiopulmonary bypass and continued to increase when MV was resumed. After 6h of MV, they were lower in patients receiving TV of 8 mL.kg^-1^ than in patients receiving TV of 10-12 mL.kg^-1^.^39^ These data illustrate the "two hit hypothesis". The lung inflammation resulting from high TV (first hit), cumulates with inflammation resulting from one-lung ventilation or cardiopulmonary bypass (second hit), which facilitates the onset of respiratory complications.

**Figure 5:**
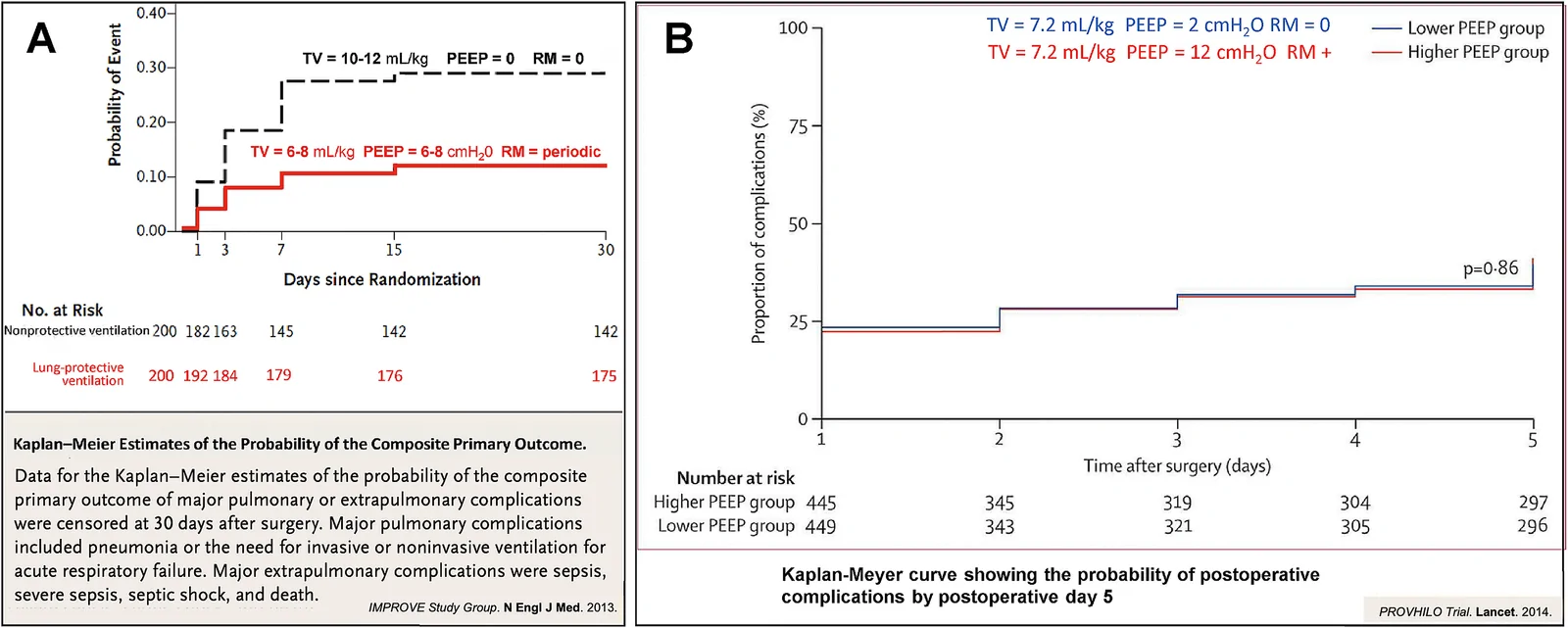
Respective effects on postoperative pulmonary complications of tidal volume (TV) and positive end-expiratory pressure (PEEP) in patients undergoing major abdominal surgery. (A) The IMPROVE multicenter randomized controlled trial randomly assigned 400 adult patients at intermediate to high risk of pulmonary complications after major abdominal surgery to either nonprotective mechanical ventilation (high tidal volume, no PEEP) or a strategy of lung-protective ventilation (low tidal volume, moderate PEEP, high-pressure recruitment maneuvers). Lung-protective ventilation reduced the risk of pulmonary and extrapulmonary complication and reduced the length of hospital stay. Reproduced from reference^40^ with permission of the publisher. (B) The PROVHILO multicenter randomized controlled trial randomly assigned 688 adult patients at intermediate to high risk of pulmonary complications after major abdominal surgery to either low PEEP (tidal volume of 7.2 mL.kg^-1^, PEEP < 2 cmH_2_O, no recruitment maneuver) or high PEEP (tidal volume of 7.2 mL.kg^-1^, PEEP of 12 cmH_2_O, recruitment maneuvers). The incidence of postoperative pulmonary complication was not affected by the PEEP level. Reproduced from reference^41^ with permission of the publisher.

In 2013 in the IMPROVE multicenter, double-blind, randomized controlled trial, 400 patients at risk of pulmonary complications after major abdominal surgery were randomly assigned to receive either non-protective mechanical ventilation (TV of 10-12 mL.kg^-1^, no PEEP and no recruitment maneuver) or lung-protective ventilation (TV of 6-8 mL.kg^-1^, PEEP of 6-8 cmH_2_O, periodic high-pressure recruitment maneuvers).^40^ The primary outcome was a composite of major pulmonary and extrapulmonary complications occurring within the first 7 days after surgery. As shown in Figure 5, the lung-protective ventilation reduced the incidence of pulmonary and extrapulmonary complications from 27.5% to 10.5% and the hospital stay by two and half days.

A subsequent RCT, where TV of 8 mL.kg^-1^ was delivered in all patients, showed no benefit to use high PEEP (12 cmH_2_O) associated to high-pressure recruitment maneuvers versus low PEEP (< 2 cmH_2_O) without recruitment maneuver.^41^ A recent RCT showed that selecting the highest PEEP according to the lowest driving pressure did not change the incidence of pulmonary and extrapulmonary complications.^42^ In both studies, intraoperative hypotension requiring the use of vasopressors was a common complication. From the mid-2010s, TV ≤ 8 mL.kg^-1^ of ideal body weight associated with moderate PEEP and periodic sigh rather than high-pressure recruitment maneuvers, became the reference for the intraoperative ventilation of patients undergoing major surgery.

The Consensus Recommendations on Perioperative Mechanical Ventilation Strategies, developed under the coordination of the Brazilian Society of Anesthesiology, have taken into consideration the evidence accumulated for more than 70 years.^1^ One of the most important recommendations is to reduce intraoperative TV to 6-8 mL.kg^-1^ predicted body weight and avoid systematic high PEEP and high-pressure recruitment maneuvers. Specificities concerning each type of surgery are detailed. Recognizing that inappropriate mechanical ventilation can contribute to postoperative pulmonary complications and affect outcomes of major surgery represents an important step toward improving the quality of anesthesiology care.

## Data availability statement

No new data were created or analyzed in this study. Data sharing is not applicable to this article.

## Conflicts of interest

The authors declare no conflicts of interest.
